# T cells, NK cells, and tumor-associated macrophages in cancer immunotherapy and the current state of the art of drug delivery systems

**DOI:** 10.3389/fimmu.2023.1199173

**Published:** 2023-06-30

**Authors:** Ya-long Yang, Fei Yang, Zhuan-qing Huang, Yuan-yuan Li, Hao-yuan Shi, Qi Sun, Yue Ma, Yao Wang, Ying Zhang, Sen Yang, Guan-ren Zhao, Feng-hua Xu

**Affiliations:** ^1^ Pharmaceutical Sciences Research Division, Department of Pharmacy, Medical Supplies Center, People's Liberation Army of China (PLA) General Hospital, Beijing, China; ^2^ Department of Biotherapeutic, The First Medical Centre, People's Liberation Army of China (PLA) General Hospital, Beijing, China; ^3^ Chinese People’s Armed Police Force Hospital of Beijing, Beijing, China; ^4^ Department of Pharmacy, Medical Supplies Center, People's Liberation Army of China (PLA) General Hospital, Beijing, China

**Keywords:** cancer immunotherapy, cancer-immunotherapy cycle, T cells, NK cells, TAM, drug delivery systems

## Abstract

The immune system provides full protection for the body by specifically identifying ‘self’ and removing ‘others’; thus protecting the body from diseases. The immune system includes innate immunity and adaptive immunity, which jointly coordinate the antitumor immune response. T cells, natural killer (NK) cells and tumor-associated macrophages (TAMs) are the main tumor-killing immune cells active in three antitumor immune cycle. Cancer immunotherapy focusses on activating and strengthening immune response or eliminating suppression from tumor cells in each step of the cancer-immunity cycle; thus, it strengthens the body’s immunity against tumors. In this review, the antitumor immune cycles of T cells, natural killer (NK) cells and tumor-associated macrophages (TAMs) are discussed. Co-stimulatory and co-inhibitory molecules in the three activity cycles and the development of drugs and delivery systems targeting these molecules are emphasized, and the current state of the art of drug delivery systems for cancer immunotherapy are summarized.

## 1 Introduction

Cancer remains a major threat to human health and life worldwide in the 21^st^ century. Cancer can be treated by surgery, chemotherapy, radiation therapy, hormone therapy, emerging immunotherapy, and targeted therapy (1). Although conventional treatments have delayed tumor progression, they have inevitable limitations including nonspecific targeting, severe side effects, and drug resistance. Cancer immunotherapy has changed the pattern of cancer treatment. It initiates and maintains the ‘cancer-immunity’ cycle by improving the immunity of our bodies or relieving the immune suppression induced by tumors, thus enhancing the antitumor immune response to control or even specifically eliminate tumors. Cancer immunotherapy has become an unexpectedly fairly effective way to fight cancer (2) such that *Science* rated it the most significant scientific breakthrough of the year in 2013.

The essence of cancer immunotherapy is to activate the immune system of the body and then produce a series of antitumor immune responses. T cells, natural killer (NK) cells, and macrophages play vital roles in cancer immunotherapy. T cells are the main members of the adaptive immune system. The surface receptors on T cells can specifically bind to tumor antigens, which activate the immune response and thus induce tumor cell death (3). As an important component of the adaptive immune system, T cells play vital roles in antitumor immunotherapy because of their high antitumor specificity and high tumor-killing potency. Chen et al. (4) proposed the concept of the cancer-immunity cycle in 2013. According to this concept, the immune cycle of T cells was divided into seven steps: (1) cancer cell antigen release; (2) cancer cell antigen presentation; (3) T cell initiation and activation; (4) T cell migration to the tumor site; (5) T cell infiltration into the tumor; (6) T cell recognition of cancer cells; (7) T cell killing of cancer cells. NK cells are an important component of the innate immune system and the first line of defense against cancer cell encroachment (5). NK cells recognize tumor cells by downregulating inhibitory receptors and upregulating stimulatory receptors, resulting in rapid and extensive killing of tumor cells (6). In 2020, Bald et al. (7) introduced the concept of the NK cell-cancer cycle where the NK cell response to cancer is divided into four steps: (1) recruitment of NK cells to the tumor microenvironment (TME); (2) recognition and activation of tumors by NK cells; (3) killing of NK cells of *in situ* and metastatic tumors; (4) coordination of adaptive immunity of NK cells. Tumor-associated macrophages (TAMs) are primarily derived from monocytes in the organism and can be polarized into two opposite phenotypes: classically activated or pro-inflammatory M1 macrophages and selectively activated, anti-inflammatory, or tissue-repairing M2 macrophages (8). The M1 macrophage is a generalist in the innate immune system. It can phagocytose and kill tumor cells, and exerts the functions of immune defense, immune self-stabilization, immune surveillance, antigen presentation. However, the TME is dominated by M2 macrophages, which suppresses the immune response and promotes tumor development. Strategies such as the elimination of TAMs, inhibition of macrophage recruitment, and repolarization of M2 into M1 will all be beneficial in reshaping the TME and upregulating the antitumor effects of TAMs.

**Figure 1:**
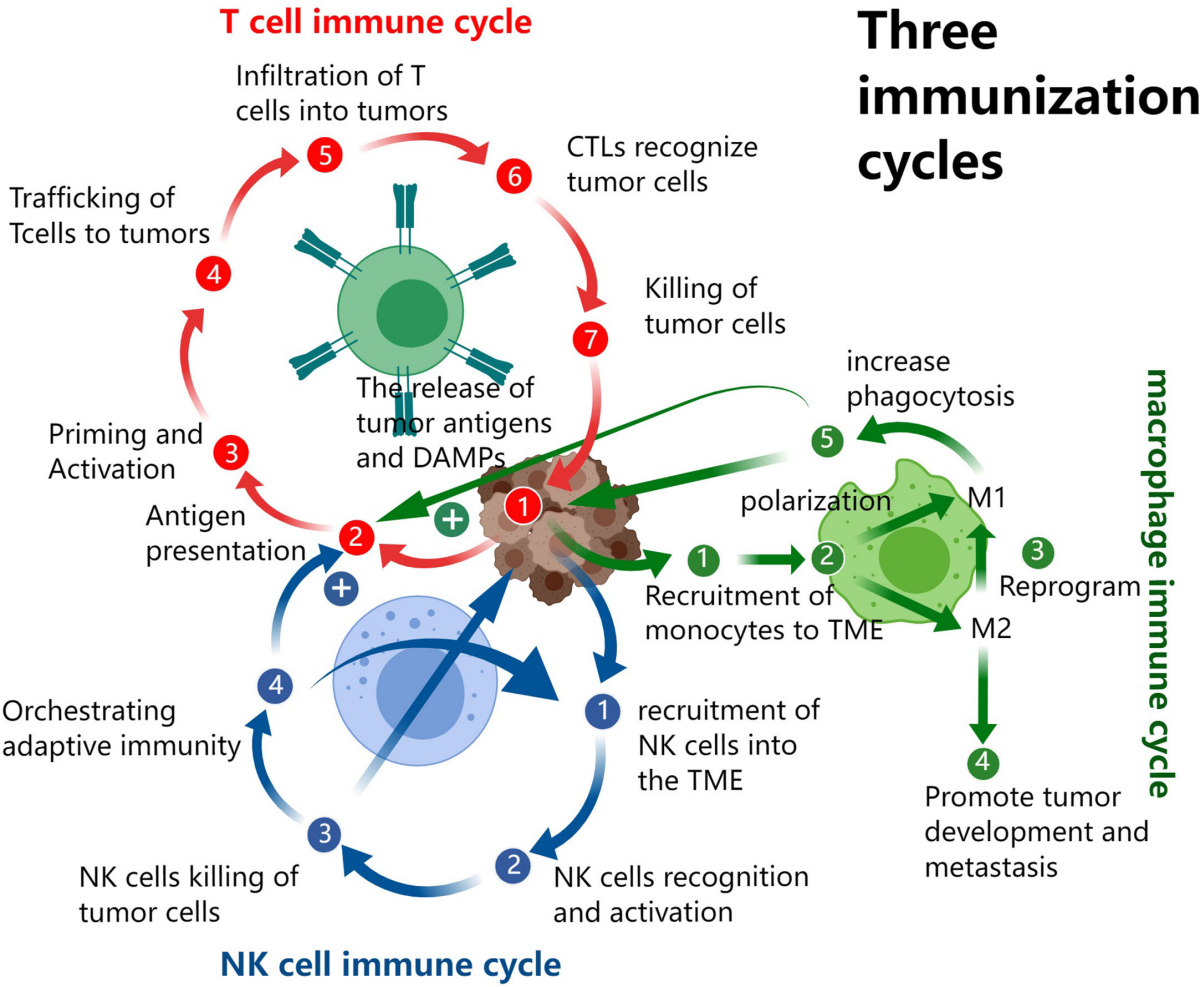
The T cell, NK cell and TAM based cancer-immunity cycles.

However, the functional mechanism of the body’s immune system is far more complex than we imagined, and the antitumor immunity of the three systems is not independent but interact with each other. Cytotoxic T lymphocytes (CTLs) kill tumor cells and thus promote the release of antigens and damage-associated molecular patterns (DAMPs). Pattern recognition receptors (PRRs) are widely present in NK, T, macrophages, antigen-presenting cells (APCs), and even in non-immune cells such as endothelial, epithelial and fibroblasts (9). After receiving the DAMPs signal, all these different cells will produce a series of immune responses; and will secrete a variety of cytokines to transmit and amplify the DAMPs signal which further recruit more NK, TAMs and dendritic cells (DC). On recognition of tumor cells, NK cells secrete cytokines such as IFN-γ, GM-CSF, G-CSF, M-CSF, TNF, IL-5, IL-10, and IL-13 to exerts immunoregulatory functions, including inhibition of tumor development, promoting DC recruitment and maturation, improving presentation of tumor antigens, as well as directly regulating activation, proliferation, and effects of T cells (7). Macrophages can recognize and phagocytose tumor cells. They also produce a variety of cytokines to promote the recruitment of NK cells, T cells, APC, and TAMs, also the presentation of antigen (10). The T cell, NK cell and TAM-based cancer-immunity cycle is illustrated in **Figure 1**.

Tumor immunotherapy includes immune cell therapy and drug immunotherapy. Immune cell therapy includes chimeric antigen receptor (CAR)-engineered T cells (CAR-T), CAR-engineered NK cells (CAR-NK), CAR-macrophages, and adoptive cell transfer (11). The key to drug immunotherapy, including monoclonal antibodies and pharmaceutical preparations based on targeted drug delivery system (2), is to activate immune cells. The activation of T cells needs not only the first signal provided by the binding of T-cell receptors (TCRs) and the major histocompatibility complex (MHC)-antigen complex, but also the co-stimulation signals provided by the APC. Maintenance of T cell activation is regulated by co-stimulatory and co-inhibitory signals (12). The activation and performance of other immune cells, such as DC, NK, and macrophages, are also regulated by co-signaling molecules. Therefore, drug immunotherapy is mainly aimed at co-signaling regulation. However, most co-signaling molecules are expressed in multiple kinds of cells, and the regulation of signaling molecules in a certain immune cell may produce responses in multiple immune cell subsets. For example, the programmed cell death protein 1 (PD-1) is expressed in T cells, NK cells, and macrophages, while its ligand, the programmed cell death ligand 1 (PD-L1), is highly expressed in tumor cells, enabling tumor cells to inhibit immune cell activity and reduce their lethality. Therefore, PD-L1 inhibitors can restore not only T cell activity, but also that of NK and macrophages (13). Another example is the Fas ligand (FasL/CD95L). FasL is expressed in T cells, APC, NK cells, and macrophages. It binds to Fas (CD95) in tumor cells to induce tumor apoptosis (14), so up-regulation of FasL expression will inhibit tumor growth. However, FasL is also expressed on the membrane of tumor cells (15). The binding of FasL of tumor cells with Fas in immune cells such as T cells, APCs, NK cells and macrophages could also induce immune cell death (16). Thus, the co-signaling regulation needs to be precise and balanced in multiple dimensions.

## 2 T cell subsets

**Figure 2:**
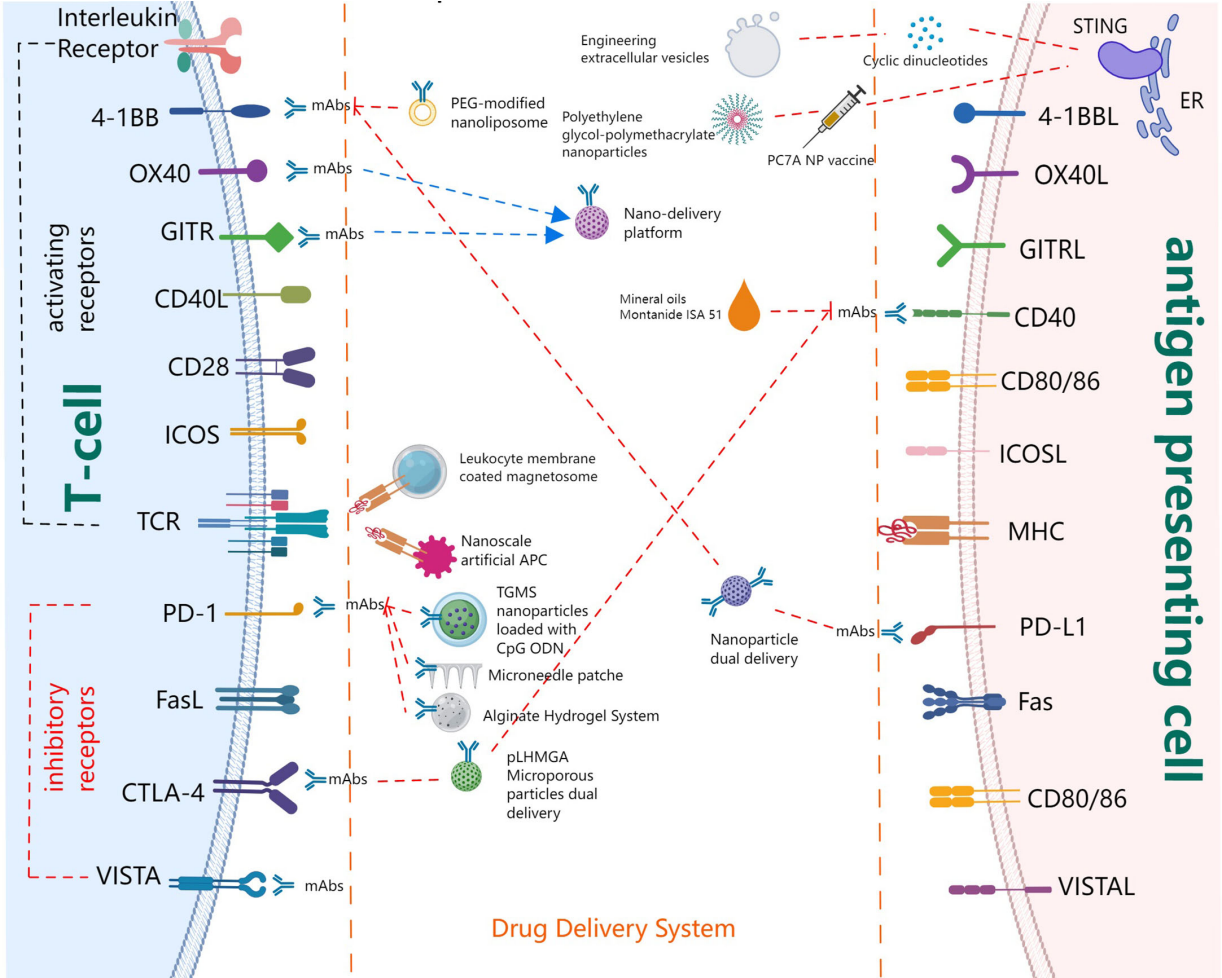
Major players in T cell co-signaling pathways and drug delivery systems.

Immune systems can recognize tumor-specific antigens and kill tumor cells or abnormal cells through the innate immune response and adaptive immune response. Adaptive immunity comprises cellular immunity mediated by T cells and humoral immunity primarily mediated by B cells (17). As the main components of lymphocytes, T cells are the main participants in cell-mediated immunity. Meanwhile, multiple cytokines including interferon (IFN) are secreted and released by T cells to expand and strengthen the T cell-mediated immune response. T cells play a key role in controlling the production, growth, and development of tumors. Furthermore, T cells can activate other components in the immune systems. By participating in humoral immunity, T cells help B cells produce antibodies and exhibit antitumor effects. The T cell is the most studied antitumor immune cell. In this section, we will focus on the initiation and activation of T cells together with its recognition and killing effect on tumor cells, that is, steps 3,6 and 7 of the T cell immune cycle in **Figure 1**. From the aspects of promoting APC to present tumor antigens, enhancing the expression of co-stimulatory receptors on T cells, and inhibiting or blocking the expression of co-inhibitory receptors, we summarize both the progress and the application status of drug delivery systems (DDSs) in these areas, as illustrated in **Figure 2**.

### 2.1 T-cell activation

APCs present tumor antigens to naïve T cells in lymph nodes and provide co-stimulatory signals. On accepting these double signals, naïve T cells become effectors. Subsequently, the downstream signaling pathways of T-cell receptors (TCRs) are initiated and a series of cascade reactions are triggered. Besides T cell activation, the expression of immune checkpoints such as PD-1 and cytotoxic T-lymphocyte-associated protein 4 (CTLA-4) on the surface of activated T cells is also up-regulated. The activation of T cells is regulated by the dynamic balance between stimulatory signals and inhibitory signals (18).

#### 2.1.1 Regulation of antigen presentation by APCs

APCs are widely distributed *in vivo*, but the total quantity is small. Nanoscale artificial APCs (aAPC) carry MHC molecules thus helping to form more MHC-antigen peptide complexes for TCR recognition, to improve the presentation of tumor antigens and promote T cell activation, which in turn stimulate tumor antigen-specific immune responses (19). Zhang et al. (20) developed biomimetic magnetosomes as versatile aAPCs, wherein magnetic nanoclusters were coated with azide-engineered leucocyte membranes and then decorated with T-cell stimuli, by which peptide-loaded major histocompatibility complex Class I (pMHC-I) and co-stimulated ligand anti-CD28 (αCD28) can be conjugated to azide-modified leucocyte membrane fragments. These nano aAPCs not only exhibited high performance for antigen-specific CTL expansion and activation but also visually and effectively guided reinfused CTLs to tumor tissues through magnetic control. Focusing on the shape of nano-APCs, Meyer et al. (21) found that nonspherical nanodimensional artificial antigen presenting cells (naAPCs) mimic the T-cell/APC interaction better than equivalent spherical naAPCs, inducing stronger, and more efficient antigen specific T-cell responses, with reduced nonspecific cellular uptake and a superior pharmacokinetic profile.

Increasing the recruitment of APCs in tumor local issues will also promote T cell activation. Some studies have shown that silica can be used to recruit APCs. Silica rods assembled spontaneously *in vivo* can form a three-dimensional (3D) microenvironment for immune cells. In a mouse model the 3D microenvironment can recruit large numbers of APCs, which continuously release inflammatory signals to promote antigen presentation, thus enhancing T cell activation, differentiation and proliferation, and ultimately trigger adaptive immune responses (22).

Immature APCs have high capacity for antigen uptake and processing, but exhibit low expression of MHC and co-stimulatory molecules on the cell surface, immature APCs are weak in antigen presentation. Therefore, promoting the maturation of APCs can enhance their antigen-presenting function and stimulate T cell activation. Nanoscale artificial antigen-coupled cells (APCs) could be designed to recognize tumor antigens and promote T cell activation. Ye et al. developed a chlorogenic acid (CHA)-encapsulated self-microemulsifying drug delivery systems (SMEDDS). This system can promote APC maturation via the mesenteric lymph node transfer pathway and further enhance T cell differentiation into effector and memory T cells. It can also suppress immune checkpoints, upregulate CD40 and CD80/86 expression, and consequently inhibit tumor growth (23).

The TME may recruit APCs but plays an inhibitory role in the function of APCs. IFN and many other cytokines can promote APC maturation and greatly enhance the antigen-presenting ability of APCs. Stimulator of interferon genes (STING), a transmembrane protein, promotes IFN secretion after its activation (24). Li et al. reported a strategy for microbubble-assisted ultrasound-guided cancer immunotherapy (MUSIC). The 2,3-cyclic guanosine monophosphate-adenosine monophosphate (cGAMP) nanocomplex is conjugated to APC-targeted microbubbles and that are efficiently delivered to the APC cytoplasmic matrix by ultrasound-guided release to activate STING. The proliferation of CD4^+^ and CD8^+^ T cells was shown to be significantly increased in the MUSIC group compared to other treatment groups (25).

#### 2.1.2 Regulation of the co-signaling receptor between APCs and T cells

The appearance of inhibitory receptors on the surface of Naïve T cells was low. After T cells were activated by dual signaling, the expression of inhibitory receptors is upregulated to avoid overactivation of T cells. Subsequently, downstream inhibitory receptors in the signaling pathway block the intracellular activation pathway, and the level of T cell activation is reduced, maintaining the overall activation level in a dynamic adjustment process. Enhancing co-stimulatory signals and blocking co-inhibitory signals will both contribute to enhance T-cell activation. Various co-signaling receptors have been found on the surface of T cells, and mainly include the CD28-B7 superfamily, the TNF receptor superfamily, and the immunoglobulin-related receptor family. Co-signaling receptors are divided into stimulatory receptors and inhibitory receptors.

CD40, or tumor necrosis factor receptor superfamily member 5 (TNFRSF5), is a co-stimulatory molecule expressed on a variety of cells, including APCs, B cells, macrophages, activated CD4^+^T cells and endothelial cells. Binding of CD40 to the CD40L ligand on the T surface activates T cells and provides an effective second signal for the onset of adaptive immunity. Potent antitumor immune responses are induced by targeting the delivery of agonist antibodies to CD40 (26). CD40 agonist antibodies have good clinical potential either as monotherapy or in combination with cytokines or chemotherapy. Fransen et al. (27) used the sustained-release additive ISA-51 as a drug carrier to deliver agonistic CD40 antibody to the lymph nodes around the tumor. The antibody stimulated CD40 molecules on the APC of local tumor tissue, activates tumor-specific CD8^+^ T cells, and is capable of eradicating local tumor tissue and distant associated tumors. No local injection sites or systemic toxicity reactions were observed, indicating its good safety profile along with good targeting ability.

TNFRSF9, or 4-1BB, or CD137, are transiently expressed on activated T cells, activated NK cells, or mature DCs (28). Its ligand 4-1BBL is expressed on the surface of APCs. Combined with 4-1BBL, 4-1BB recruits TNF receptor-associated factor (TRAF), activates intracellular signals such as NF-κB and mitogen-activated protein kinase (MAPK), induces activation and proliferation of T cells, and promotes the production of interleukin (IL)-2 and IFN-γ. The T cells are then differentiated towards memory T cells and effector T cells. Meanwhile, co-stimulatory CD28 signals significantly improved the expression of 4-1BB (29). Kosmides et al. combined PD-L1 antibody inhibitors with 4-1BB through a nanodelivery platform. By blocking PD-L1 and stimulating 4-1BB, increased infiltration of CD8^+^ T cells in tumor tissue was observed in a mouse model of melanoma and colon cancer. This combination was superior to PD-L1 or 4-1BB used alone to inhibit tumor growth and prolonged survival in mice (30).

CTLA-4, also known as CD152, a member of the immunoglobulin-associated receptor family, is a protein receptor expressed in T cells shortly after T cell activation and is a key negative regulator of T cell function (31). As a homolog of the co-stimulatory molecule CD28, it binds with higher affinity to the CD80 and CD86 ligands on the APC, blocking the co-stimulatory signal and transmitting the inhibitory signal to T cells. Li et al. prepared nanoparticles (NPs) encapsulating CTLA-4 siRNA, using biocompatible and biodegradable polyethylene glycol blocks (poly d, l-lactide) as the coupling agent, which could enter T cells both *in vitro* and *in vivo*. After entering T cells, CTLA-4 siRNA silence CTLA-4 mRNA and inhibited CTLA-4 expression on the surface of T cells to enhance T cell activation and proliferation. In the range of 200 nmol/L to 400 nmol/L, CTLA-4 siRNA significantly reduces CTLA-4 mRNA levels in activated T cells dose-dependently after 48 h of treatment (48.7 ± 4.9% and 33.1 ± 4.6%, respectively) (32).

### 2.2 Regulation of T-cell cytotoxic effect

Naïve T cells differentiate into CD8^+^ T cells after activation and migrated to the tumor site. Subsequently, CD8^+^ TCR specifically combine with the MHC-I molecule-tumor antigen complex, and CTL begin to recognize and kill tumor cells and promote their proliferation and differentiation. Cytokines such as IL-2/IL-12 may improve CD8^+^ T cell differentiation and proliferation. CD8^+^ T cells promote tumor cell death and lysis by secreting perforin, granzyme, IFN, TNF and other mediators, and also promote tumor cell apoptosis by combining FasL with Fas receptor on the tumor cell surface (33, 34).

Due to the high expression of inhibitory ligands such as PD-L1 and the low levels of co-stimulatory ligands on the surface of tumor cells, when tumor cells bind to CD8^+^ T cells, they enhance the inhibitory signal and diminish or even inhibit the killing capacity of T cells, thus helping tumors to evade CTL killing. Therefore, blocking inhibitory signaling pathways or strengthening the stimulatory signal could restore the cytotoxic effect of CTL cells. Co-inhibitory receptors mainly include PD-1, CTLA-4, galectin-1 (Gal-1), V-domain Ig suppressor of T cell activation (VISTA), T cell immunoreceptor with immunoglobulin and ITIM domains (TIGIT), while co-stimulatory receptors mainly include glucocorticoid-induced TNFR-related protein (GITR), TNFRSF4 (also known as OX40), 4-1BB, CD28, and CD40, as shown in **Figure 2**.

#### 2.2.1 Blocking co-inhibitory signals

PD-1, also known as CD279, belongs to the B7-CD28 superfamily and is expressed in activated T cells and other cells. PD-1 has two known ligands belonging to the B7 family, B7-H1, or PD-L1, also known as CD274, and B7-DC, or PD-L2, also known as CD273 (35). PD-1 or PD-L1 inhibitors interrupt the binding of PD-1 in activated T cells to PD-L1 in tumor cells and block negative immune regulation, can thus, restore T cell-mediated antitumor immune responses (36). Despite some excellent clinical results, PD-1/PD-L1 antibody agents still face challenges such as low response rates, low binding intensity and uncontrollable side effects, while small molecule inhibitors also face unsuitable rapid clearance rates and low tumor site accumulation (37). To improve the therapeutic efficacy of PD-1/PD-L1 inhibitors, nanotechnology-based DDS have been gradually introduced to achieve tumor-targeting drug delivery to enhance the interruption of the PD-1 and PD-L1 combination (38). Tumor targeting can also reduce off-target effects that would lead to autoimmune disease or systemic side effects (39). Wang et al. (40) developed an innovative delivery carrier for the controlled release of loaded aPD1 and unmethylated cytosine-phosphate-guanosine oligodeoxynucleotides (CpG-ODN) in response to inflammation. The carrier (designated as DNA ‘nano-cocoons’, DNCs) is assembled by a long-chain single-stranded DNA (ssDNA), with repeated interval CpG sequences and harboring cutting sites of restriction enzyme HhaI that are capable of digesting DNCs and subsequently generating CpG ODN fragments. HhaI is captured into triglycerol monostearate (TGMS) NPs (TGMS NPs) and attached to DNCs. TGMS is an amphiphile that could be cleaved by esterase and matrix metalloproteinases (MMPs) that are highly expressed at the wound sites and are suitable for developmental tissue remodeling. Triggered by the inflammatory condition that occurs at the wound site of the tumor resection incision, TGMS can be enzymatically cleaved, which disassembles the cage and releases HhaI, which can further sequentially convert DNCs to CpG ODNs and release aPD1. In a melanoma model, the combined action of sustained released CpG ODNs and aPD1 can synergistically facilitate induction of durable and specific antitumor T-cell responses, which in turn substantially improve the anticancer immune response to treat remaining or metastatic tumors after resection of primary tumors. In addition to NPs, new DDS based on self-degrading microneedle patches (41), alginate hydrogel systems (42) and targeted cationic liposomes (43) are also adopted to deliver PD-1 antibodies to target the PD-1/PD-L1 axis in order to exert inhibitory or blocking effects and provide better antitumor therapeutic efficacy of PD-1/PD-L1 inhibitors.

Generally, only small amounts of CTLA-4 can be detected on the cell surface of resting T cells in general, as most CTLA-4 molecules are located in intracellular compartments of nuclear Golgi vesicles, endosomes and lysosomes. When T cells are activated by co-stimulation of TCR/CD28, CTLA-4 is induced to relocate to the cell surface. CTLA-4 plays a crucial role in the controlling T cell activation and tolerance. On one hand, it mediates the immunosuppressive capacity of Tregs; and on the other, it expresses CTLA-4 after resting T cell activation, acting as a negative feedback signal to balance the strength of the adaptive immune response. Li et al. (32) prepared NPs encapsulating CTLA-4 siRNA, which delivered CTLA-4 siRNA to CD4^+^ and CD8^+^ T cell subsets in tumor tissue. The NPs inhibited CTLA-4 expression on the surface of T cells, significantly increased the percentage of active CTL cells, and decreased the proportion of inhibitory regulatory T cells (Tregs). CTLA-4 siRNA NPs significantly prolonged mouse survival in a dose-dependent manner, the median survival of mice loaded with B16 melanoma was extended from 25 days (PBS treatment) to 32 days with a 1 mg/kg of siCTLA-4 NPs injection, and to 42 days with 2 mg/kg doses.

Galectin-1(Gal-1) is a member of the glycoconjugate family of proteins which is highly expressed on the surface of tumor cells. Gal-1 promotes the differentiation of tolerogenic dendritic cells, the apoptosis of effector T cells and the proliferation of Tregs in the TME, and has been found to induce immune tolerance and further escape of tumor cells from the immune response in a variety of tumors (44). Woensel et al. developed Gal-1 siRNA-loaded chitosan NPs. Chitosan NPs protect Gal-1 siRNA from RNA enzyme degradation. Gal-1 siRNA was delivered by nasal administration, to mouse glioblastoma multiforme (GBM) to interfere with the expression of Gal-1. More than 50% reduction in Gal-1 expression was observed in GBM-bearing mice, which induced a significant shift in the composition of the TME with a decrease in myeloid suppressor cells and regulatory T cells as well as an increase in CD4^+^ and CD8^+^ T cells. As a result, a significant increase in the mouse survival rate was obtained (45, 46).

VISTA is a newly identified immune checkpoint that regulates a wide range of immune responses. VISTA has a structure similar to that of PD-L1. Both are potent suppressors of T cell function. However, unlike PD-L1 up-regulating after T cell activation, VISTA is an inhibitory receptor expressed on naïve T lymphocytes, which has the ability of pre-negatively adjustment (47). The drug CA-170 is an oral small molecule dual antagonist that selectively targets PD-L1 and VISTA. CA-170 exhibits superior antitumor effects in multiple tumor models and has a good safety profile (48).

#### 2.2.2 Enhancing co-stimulatory signal

Tumor necrosis factor receptor superfamily 18 (TNFRSF18, CD357), also known as GITR, is an important target for tumor immunotherapy. GITR activation blocks Treg suppression and enhances T cell proliferation, thus exerting tumor-killing activity. DTA-1 is an agonist monoclonal antibody against GITR (49). *Kelsey et al.* evaluated the efficacy of DTA-1 antibodies in treating esophageal squamous cell carcinoma (ESCC). In mouse models, the CTL/Treg ratio in the esophageal mucosa of mice treated with the carcinogen 4-nitroquinoline 1-oxide (4-NQO)/DTA-1 expression was significantly higher than that obtained following treatment with 4-NQO/IgG (50).

TNFRSF4 (OX40 CD134), is type I transmembrane glycoprotein that is initially expressed by Tregs and, upon activation, by effector T cells. It plays role upon binding to the OX40L ligand. OX40 binds to factors related to the TNF receptor (TNFR) involved in signal transduction pathways, which can mediate nuclear factor κ light chain (NF-κB) activation of B cells, induce the expression of proteins with antiapoptotic activity or that inhibit the cell cycle, counteracts the suppressed state of T, B, and NK immune cells, and simultaneously stimulates T cells in effector T cells (51). Chen et al. (52) developed a biodegradable poly (lactic-co-glycolic acid) (PLGA) nanoparticle loaded with OX40 antibody with an average particle size of 86 nm. The drug loading rate is 25%.

Compared to CTLs treated with anti-OX40 monoclonal antibody or PLGA NPs alone, CTLs treated with anti-OX40 PLGA NPs have a significantly higher percentage of proliferation. The multitarget delivery system was designed to carry specific antibodies that simultaneously target tumor cells and CTLs, so as to guide the tumor-orientated killing effect of CTL. For example, blinatumomab (53) links the CD3 protein complex on the T cell surface and CD19 on the surface of tumor cells to accelerate the lyse of tumor cells by TCL. The 5-year overall survival data from Amgen for patients with minimal residual disease (MRD)-positive acute lymphoblastic leukemia (ALL), showed that at a median follow-up period of 59.8 months, the median overall survival reached 36.5 months. Other bispecific antibodies include amivantamab, an EGFR/c-Met bispecific antibody for non-small-cell lung cancer (NSCLC) treatment, and tebentafusp, a novel gp100×CD3 for treatment of HLA-A*02:01 positive non-resectable or metastatic uveal melanoma. Based on bispecific antibody technology, Wu et al. (54) developed a new type of tri-specific antibody consisting of a CD38 antibody, CD3 antibody, and CD28 antibody. The CD3 antibody and the CD28 antibody target T cells and promote T cell activation, while the CD38 antibody targets to myeloma cells, as well as certain lymphomas and leukemias. This tri-specific antibody inhibited myeloma growth in humanized mouse models and stimulated memory and effector T cell proliferation. It also reduced Tregs in nonhuman primates at a well-tolerated dose.

## 3 NK cell subsets

NK cells are cytotoxic lymphocytes of the innate immune system that simultaneously possess both innate and adaptive immunity, and contribute to protecting the host by killing infected, mutated, senescent, damaged or invasive cells (55–57). NK cells kill tumors nonspecifically and directly, without the need to recognize tumor antigens or the involvement of antibodies, and are free from the restriction of MHC, playing an important role in antitumor immune responses in the TME. when encountering tumor cells, resting NK will be activated to exert a killing function via adhesion, granule polarization, degranulation, and cytokine secretion.

**Figure 3:**
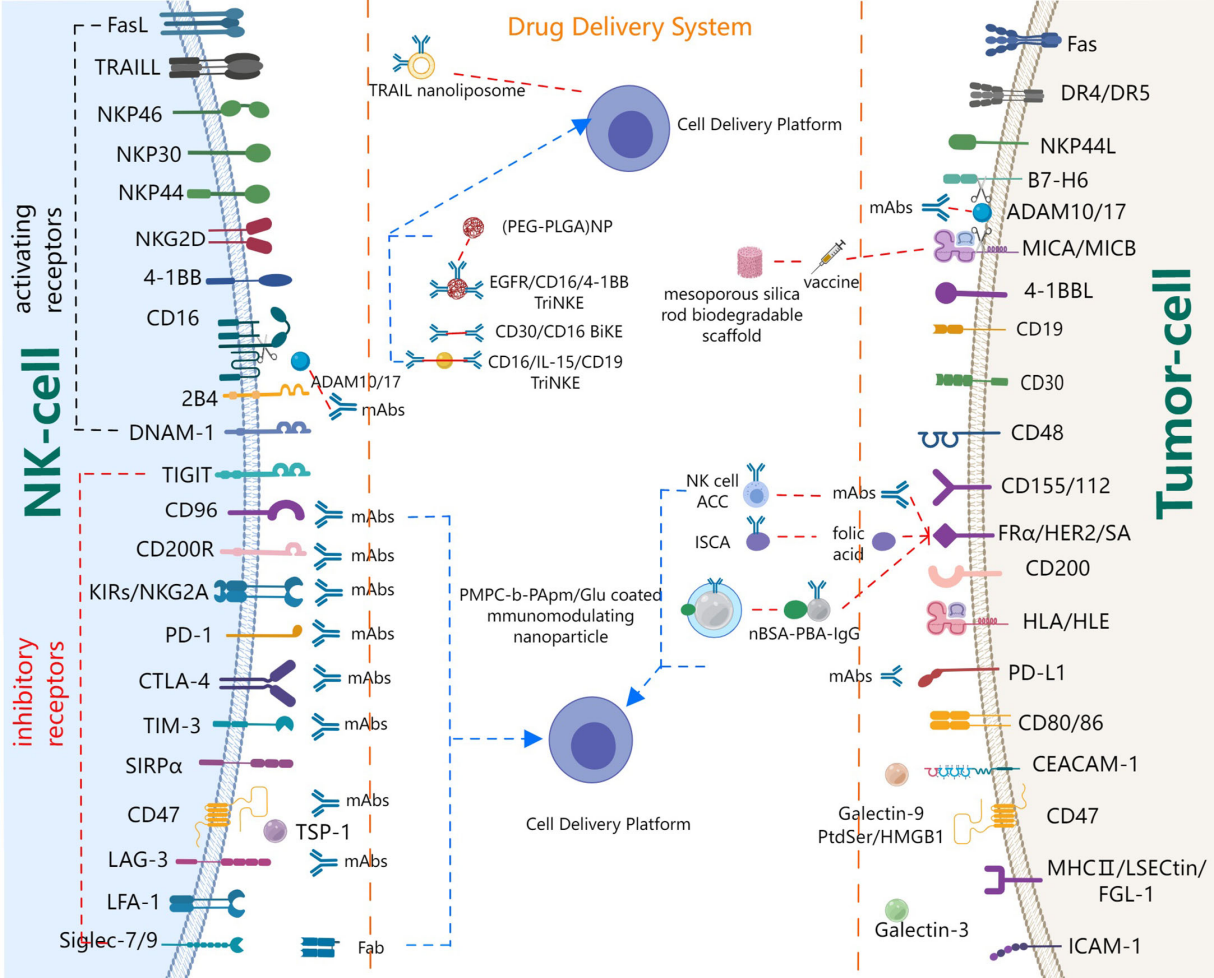
Major players and drug delivery systems of NK cell co-signaling pathways.

Bald et al. (7) proposed the concept of the NK cell-cancer cycle, demonstrating the importance of NK cells in cancer immunotherapy. In this review, we will focus on receptors and cytokines, as well as related targeted delivery systems that regulate NK cell recognition, activation, and cytotoxicity, as summarized in **Figure 3**. Based on their effects on NK cell activation, receptors on resting NK cells could be roughly divided into two categories: inhibitory receptors and stimulatory receptors (58). Stimulatory receptors are of three types. The first class of receptors is a cell surface activation complex that is composed of different transmembrane ligands and transmembrane adapter proteins, such as killer activating receptor-associated protein/DNAX activating protein of 12 kDa (KARAP/DAP12), FcRγ, CD3ζ, and DAP10 (58). Of these, KARAP/DAP12, FcRγ, and CD3ζ are polypeptides carrying immunoreceptor tyrosine-based activation motif (ITAM). Therefore, the first class of receptors is also called ITAM-carrying receptors, for example, CD16, NK p46, NK p44. The signaling pathways that lead to cytokine secretion appear to be strictly dependent on ITAM-carrying receptors. The second class of receptors is ITAM-independent receptors. One of the most studied ITAM-independent receptors is 2B4, which plays a complex role. Several studies have shown that 2B4 is an activating receptor on resting NK cells, which was still a key inhibitory receptor during NK cell development (59–61). The third class of receptors are integrins, mainly including lymphocyte function-associated antigen-1 (LFA-1) (58), macrophage-1 antigen (Mac-1, CD11c/18) (7), very late antigen-4 integrin (VLA-4; CD49d/CD29), VLA-5, which can help enhance binding between NK cells and target cells. Meanwhile, some pattern recognition receptors, such as Toll-like receptors (TLR), NOD-like receptors (NLR), retinoic acid-inducible gene I (RIG-I)-like receptors (RLR), C-type lectin receptors (CLR) and multiple intracellular DNA sensors (62), are also beneficial to enhance activation signals. Inhibitory receptors mainly include KIR inhibitory receptors and common immune checkpoints.

### 3.1 Recognition of tumor NK cells and activation

NK cells identify tumor cells and healthy cells primarily by whether the signals delivered by activating receptors and by inhibitory receptors are balanced (63). There are mainly three mechanisms for identification: missing-self-identified, induced self-ligands, and antibody-dependent cell-mediated cytotoxicity. Most tumor cells are considered ‘non-self’ by NK cells due to their reduction or deficiency of MHC-I, which is expressed in almost every healthy cell *in vivo*. Many tumor cells downregulate the expression of MHC-I in order to evade detection by cytotoxic CD8^+^ T cells (64), while this meets the recognition needs of NK cells and is beneficial for NK cells to kill escaped tumor cells as a cooperation mechanism with CTLs to fight tumors. Tumor cells typically up-regulate stimulatory ligands for NK cell activating receptors (63, 64). This tilts the overall balance in favor of the activation signal, leading to strengthening NK cell cytokine release and cytotoxicity against the tumor cell. Furthermore, after recognizing and binding to the tumor antigen, the tumor antigen-specific antibody binds to CD16 NK cell surface receptor, which can also enhance the activation signal of NK and trigger antibody-dependent cytotoxicity (64).

### 3.2 Killing of tumor cells by NK cells

The main killing mechanisms of NK cells include the following mechanisms (7, 65, 66): (1) NK cells release killer mediators to kill target cells; (2) NK cells kill tumor cells through granule exocytosis; (3) NK cells induce apoptosis of tumor cells; (4) NK cells induce pyroptosis of tumor cells; and (5) NK cells kill tumor cells through antibody-dependent cell-mediated cytotoxicity. Various receptors in NK cells, together with their ligands, jointly regulate effector function and cytokine secretion of NK cells. Inhibitory receptors and activating receptors on resting NK cells regulate the efficacy killing mechanism 1, 2, and 5. NK cells induce apoptosis mainly through the death receptor signaling pathways Fas, TNFR1, and tumor necrosis factor (TNF) related apoptosis-inducing ligand (TRAIL). Recent studies have shown that NK could also induce cell death through pyroptosis, mainly by the gasdermin B molecule (GSDMB), which is a common pro-inflammatory cell death in innate immunity.

Multiple cytokine signals performed via the janus kinase/signal transducer and transcription activator (JAK/STAT) pathway to orchestrate NK cell development and maturation. Natural killer cell stimulatory factor (NKSF), such as IL-2, IL-12, IL-15, IL-18, IL-21, IFN-I, TNF-α, and TNF-p (LT), has a booster effect on NK cells and positively regulates NK cell activation and differentiation (7, 67). At the same time, TNF can also activate the apoptosis pathway in tumor cells, like FasL. However, there are also cytokines that inhibit NK cell activation and differentiation, for example, IL-37, transforming growth factor-β (TGF-β), activin-A, prostaglandins (PG) E1, E2, D2, and adrenocortical hormone.

### 3.3 Application of DDSs in the regulation of anti-tumor cytotoxic effects for NK cells

The essential role of NK cells in both innate and adaptive immunity makes NK cell-based immunotherapy a promising strategy in cancer therapy. To improve the cytotoxicity of NK cells, it is necessary to enhance the recognition and activation of NK cells, especially to increase the killing efficiency of NK cells. To enhance killing efficiency, novel DDSs were designed to load different antibodies or factors together, one end of which targets the receptors on the surface of NK cells, while the other end targets the antigens on the surface of tumor cells, specifically to bring NK and tumor cells closer. This strategy causes tumor cells to die immediately after activation of NK cells. A third activation antibody or cytokine can also be encapsulated in DDS to enhance the cytotoxic effect of NK cells.

The present antibodies primarily target activating or inhibitory receptors. In most cases, NK activation requires the coordination of different co-stimulatory receptors. CD16A (FcγRIIIA), a low affinity receptor of IgG Fc fragments, appears to be the only receptor capable of autonomously activating NK cells. Increasing the density of CD16A on the surface of NK cell membranes will significantly enhance the killing effect of NK cells (64). Monoclonal antibodies targeting NK inhibitory signal receptors mainly include killer immunoglobulin-like receptors (KIRs) inhibitors (68–70), such as lirilumab (also called IPH2102, BMS-986015) targeting KIR2DL-1, KIR2DL-2, and KIR2DL-3, lacutamab (IPH4102) targeting KIR3DL-2, NKG2A inhibitor monalizumab (IPH2201) and immune checkpoint inhibitors (ICI). In addition to commonly used anti-PD-1 and anti-PD-L1 monoclonal antibodies, ICI includes anti-CD96, anti-TIGIT, anti-T cell immunoglobulin and mucin-containing molecule 3 (TIM-3), anti-lymphocyte activation gene-3 (LAG-3), anti-CD200R, anti-CD47 monoclonal antibodies, and Fab fragments that block Siglec-7 and Siglec-9 (68, 69, 71, 72). These ICI antibodies or fragments can target NK cells and enhance the cytotoxic effect of NK cells, while at the same time relieve tumor inhibition in T cells, B cells, and macrophages. Typical tumor antigens include CD19 and HLA class II in B cell malignancies, CD30 in Hodgkin lymphoma, epidermal growth factor receptor (EGFR) in various epithelial cancers, HER2 in breast cancer, and CD33 in acute myeloid leukemia (AML) (63). NK cell receptors include CD16, NKp30, NKp44, NKp46, and NKG2D (7). Therefore, there are many combinations of targeted delivery systems.

Reusch et al. (73) constructed a tetravalent bispecific CD30/CD16A tandem diabody (TandAb^®^) consisting solely of Fv domains. The TandAb has two binding sites for CD16A and two for CD30, the antigen that specifically identifies Hodgkin lymphoma cells. The binding and cytotoxicity of TandAb were compared with antibodies with identical anti-CD30 domains: (1) native IgG, (2) optimized IgG for binding to Fc receptors, and (3) bivalent bispecific CD30/CD16A diabody. Due to its CD16A bivalence and reduced koff, TandAb was retained longer on the surface of NK cells than the IgG or diabody. This contributed to the higher potency and efficacy of TandAb compared to those of other anti-CD30 antibodies. Au et al. (74) constructed a nanoparticle-based trispecific NK cell engager (nano-TriNKE) platform. The nanoengager platform is based on the biocompatible poly (ethylene glycol)–block-poly(lactide-co-glycolide) (PEG-PLGA) NP, which is functionalized with cetuximab (anti-human EGFR antibody, α-EGFR) and two NK-activating agents: anti-CD16 (α-CD16) and anti–4-1BB (α-4-1BB) antibodies. In the mouse model of tumors overexpressed with EGFR A431, compared to the control groups of each antibody, treatment with free α-EGFR and α-CD16/α-4-1BB NPs or α-EGFR NPs, and α-CD16/α-4-1BB NPs led to moderate delays in tumor growth (P=0.0046 and 0.0061 versus the nontreatment group). Treatment with α-EGFR/α-CD16/α-4-1BB NPs had the most robust treatment responses with tumor growth delays averaging 24 days after initial treatment and prolonged survival averaging 18 days compared to the nontreatment group (P=0.0018). Cheng et al. (75) constructed a tri-specific killer conjugate (161519) consisting of anti-CD16, IL-15, and anti-CD19, which enhanced NK cell activation, proliferation, and antitumor cytokine production. It significantly enhanced NK cell cytotoxicity against CD19 tumor cells. Currently, IPH6101/SAR443579 is the first NKp46/CD16-based NK cell engager (NKCE) using Innate’s proprietary multispecific antibody format ANKETTM in a phase I/II clinical trial (NCT05086315) in various blood cancers, such as relapsed or refractory acute myeloid leukemia (R/R AML), B-cell acute lymphoblastic leukemia (B-ALL) or high risk-myelodysplastic syndrome (HR-MDS). In the study by Li et al., ACE1702 are the trastuzumab-armed oNK cell line (oNK) treated with gamma irradiation and cryopreservation. Compared to Ctrl-oNK cells without mAb, ACE1702 showed excellent potency against HER2-expressing cancer cells *in vitro* and *in vivo*, and enhanced IFN-γsecretion, which exhibited a similar effect as CAR-NK (76).

Due to the lack of an Fc region, NK cell-targeted bispecific antibodies (BiKE) and TriNKE are structurally instable and have short *in vivo* half-lives of (77). One solution is to use circulating cells as carriers loading BiKE and TriNKE. Anselmo et al. (78) found that erythrocyte-adsorbed NPs increased blood persistence by approximately 3 times and increased accumulation in the lung by approximately 7 times. They also enhanced lung-liver and lung-spleen nanoparticle accumulation by more than 15- and 10-fold, respectively.

## 4 Tumor associated macrophages

TAMs are the most abundant population type of tumor-infiltrating immune cells found in the TME (79), therefore they are the most important immune cells regulating tumor proliferation. TAMs play an essential role in innate and adaptive immunity, acting as key links in tumor immune regulation, controlling tumor growth, proliferation, and metastasis and directly affect the treatment outcome of tumor immunotherapy.

Originally, macrophages were composed of tissue resident macrophages and blood-derived macrophages (80). Tissue resident macrophages are derived from primitive yolk sac precursor cells and have tissue-specific phenotypes and functions. They play key role in metabolism regulation and mediating inflammatory immunity (81). Colony stimulating factor 1 (CSF-1) is very important for macrophage survival, differentiation and proliferation. Infiltrating macrophages are formed by bone marrow-derived peripheral blood monocyte precursors recruited locally and differentiated in response to local growth factors and chemokines, such as CCL2 and CCL5. Infiltrating macrophages are shorter lived and require constant replenishment by circulating monocytes (82). The immunosuppressive TME disrupts the physiological functions of antigen presentation and cytotoxicity of newly recruited monocytes. Compared to normal tissue homeostasis, cancer tissues are characterized by increased proliferation of tissue resident macrophages and increased monocyte recruitment (83).

Macrophages are a group of cells with high flexibility and heterogeneity, which can be polarized into different subtypes in the TME: classically activated macrophages (M1 type) and alternatively activated macrophages (M2 type) (84) according to the status and function of activation, M1 type macrophages play important roles in innate responses against invading pathogens, whereas activated M2 type macrophages are important in tissue repair and tumor progression (85). Lipopolysaccharide (LPS), IFN-γ and granulocyte-macrophage colony-stimulating factor (GM-CSF) can promote monocyte differentiation into M1 macrophages that secrete antimicrobial molecules and pro-inflammatory cytokines, including reactive oxygen species (ROS), nitric oxide (NO), and IL-6 (86). M1 macrophages are capable of directly phagocytosing tumor cells and at the same time maintaining robust antigen presentation ability. By increasing IL-12 production, M1 macrophages also boost the type 1 helper T (Th1) cell immune response to kill microbial pathogens and tumor cells (87, 88).

M2 macrophages comprise various subtypes based on expression differences of relevant phenotypic markers. For example, M2 macrophages include four subtypes M2a, M2b, M2c, and M2d in the mouse (89). M2 macrophages secrete high levels of cytokines IL-1Ra, IL-4, IL-10, and IL-13, as well as the chemokines CCL17, CCL18, CCL22, and CCL24. They also highly express Arg-1, YM-1, dendritic cell-specific intercellular adhesion molecule-3-grabbing non-integrin (sDC-SIGN), CD163, CD206, CXCR1, and CXCR2, and produces less NO and IL-12. Among these, CCL18 can promote tumor cell infiltration and metastasis. Up-regulated expression levels of CCL22 can inhibit T cell proliferation and activity, which promote tumor cell growth. sDC-SIGN plays a role in helping escape immune surveillance. Arg-1 stimulates tumor cell proliferation by accelerating arginine metabolism to generate ornithine and polyamines. Upregulating CD206 expression in macrophages may reduce the ability to generate NO, to reduce pathogen killing and clearance of pathogens (90).

**Figure 4:**
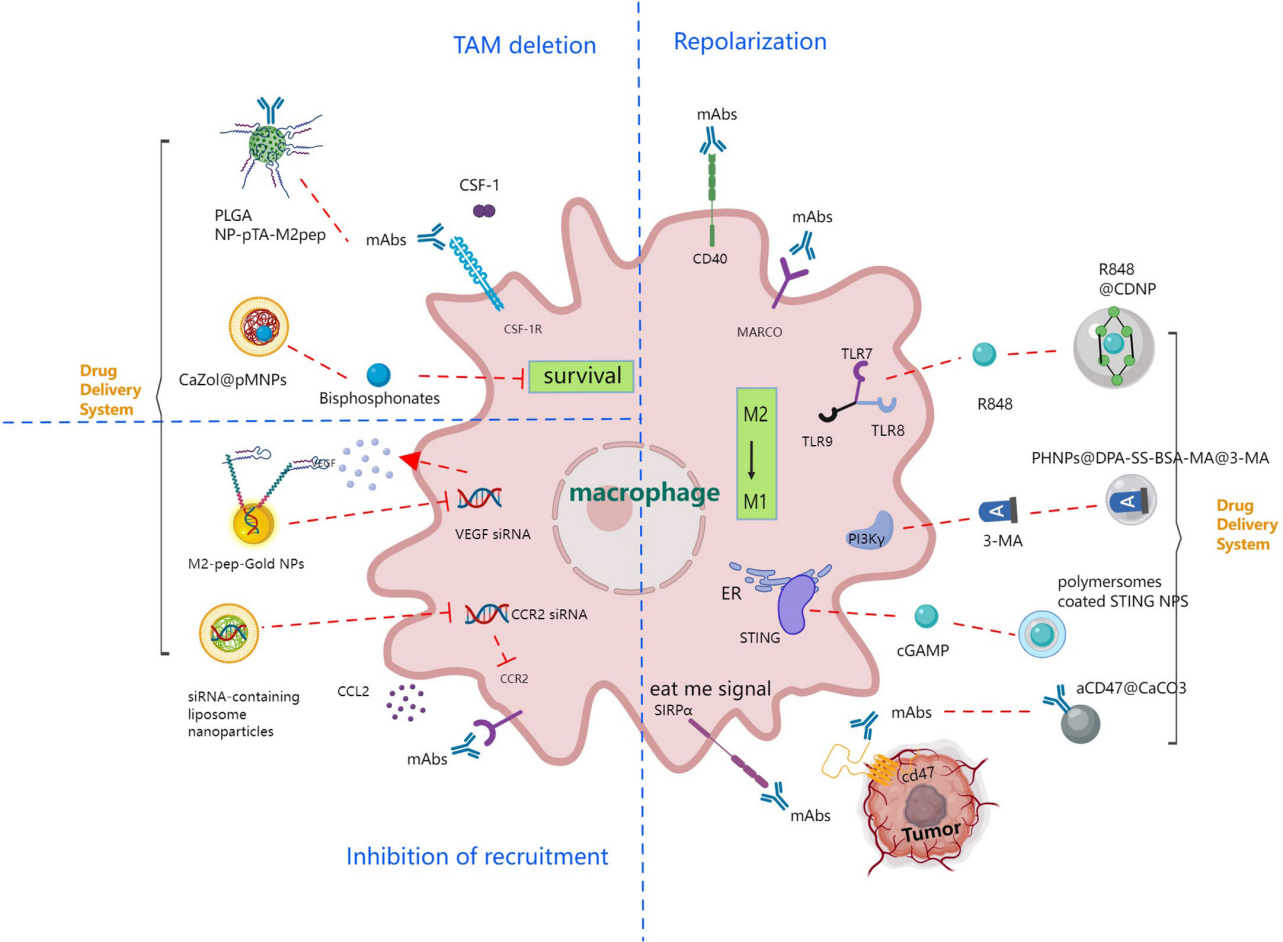
Immune strategies targeting tumor associated macrophage signaling pathways leading to TAM deletion, inhibition of recruitment and TAM repolarization.

TAMs are mainly M2 macrophages. Due to the extremely rapid proliferation and expansion of tumor tissue, the TME is in a state of hypoxia. After macrophages are recruited to the tumor area, several intracellular signaling pathways, such as the hypoxia inducible factor (HIF) pathway, the vascular endothelial growth factor (VEGF) pathway, and the nuclear transcription factor (NF-κB) pathway, are activated in the hypoxic microenvironment, leading to the accumulation of VEGF and eosinophil chemokines (Eotaxin) in tumor tissue that causes M2 polarization (91). The tumor-promoting effects of M2 TAMs manifest in multiple aspects: (1) suppression of T-cell-mediated tumor immune responses (92); (2) promotion of tumor angiogenesis (93); (3) induction of tumor migration, invasion, and metastasis (94); (4) improvement of cancer cell resistance to chemotherapy and radiotherapy (95). Therefore, based on the function of TAMs in tumorigenesis and development, targeted immunotherapeutic strategies against TAMs mainly include TAM depletion, termination of TAM recruitment, TAM repolarization (96), and improving phagocytosis, as depicted in **Figure 4**.

### 4.1 Elimination of macrophages in the tumor microenvironment

Limiting the number of TAMs within tumors can be achieved by either eliminating existing TAMs or inhibiting further Tam recruitment. The most well-established method to reduce TAM survival is to block the CSF-1 or CSF-1R axis which is crucial for macrophage differentiation and survival. CSF-1 is a master regulator and chemokine of most macrophage populations. Its receptor, CSF-1R, is a tyrosine kinase that promotes monocyte and macrophage survival, proliferation, and differentiation (97). The dependence of macrophages on CSF-1/CSF-1R signaling makes CSF-1R a target for selective depletion of TAMs. Ries et al. (98) developed a monoclonal antibody (RG7155) that inhibits CSF1R activation. The *in vitro* results showed that RG7155 induced macrophage apoptosis in TME. Pang et al. (99) designed poly (lactic-co-glycolic acid) NPs, which were coated with M2pep, a selective binding peptide ligand to M2-polarized macrophages, via a simple surface modification method based on a tannic acid-iron complex. M2pep-coated NPs can deliver PLX3397, CSF-1, or CSF-1R pathway inhibitor, for the treatment of B16F10 tumors. In Both *in vivo* and *in vitro* studies, M2-pep-modified NPs exhibited greater efficiency in the ingestion of TAM. PLX3397 encapsulated in M2pep coated NPs and attenuated tumor growth better than the free drug counterpart.

Recently, studies have found that bisphosphonates (clodronate and zoledronate), can influence the proliferation and induce apoptosis of TAMs, reduce neoangiogenesis, and eventually inhibit tumor growth and metastasis. However, its antitumor activity is often limited by its short plasma half-life and off-target effects (100). Zang et al. (101) developed lipid-coated calcium zoledronate NPs containing conjugated mannose, which specifically targeted and depleted TAMs. The NPs inhibited tumor growth with more than 70% reduction in tumor volume, led to more than 80% reduction in angiogenesis, and thus exposed the weakened immunosuppressive effects of TAMs. Folate receptor β (FRβ) is a highly expressed surface receptor on macrophages, which has a high affinity for folic acid at the nanomolar level. Therefore, folic acid modified NPs may mediate targeted delivery to TAMs (102). Hattori et al. (103) evaluated the targeting ability of zoledronic acid loaded folate conjugated liposomes (FL-ZOL). They found that FL-ZOL exhibited high cytotoxicity against murine derived macrophage RAW264.7 without any toxicity to FRβ-negative mouse colorectal adenocarcinoma CT26 cells. The results indicated that FL-ZOL could be selectively internalized by TAMs via FRβ mediated endocytosis; hence, consuming tumor-related macrophages and inhibiting tumor growth.

### 4.2 Inhibition of TAMs recruitment

TAM monocytes or macrophages are recruited from the blood and infiltrate the tumor. Several studies have determined that chemokines and ligands in the TME play a prominent role in the regulation of TAMs (104), indicating that chemokines (CCL2, CCL3, CCl4, and CCL5), CSF-1, and VEGF are potential therapeutic targets (105, 106). Therefore, restraining chemokine secretion by targeting and impacting monocytes can effectively inhibit TAM recruitment, reduce TAM infiltration in solid tumors, and reshape the immunosuppressive TME. Using small molecule inhibitors or specific antibodies to block related signal pathways is an effective way to inhibit TAM recruitment. In the mouse breast tumor model, 2.5×10 (6) MDA-MB-231 or 10 (6) LM2 tumor cells were orthotopically injected into the inguinal mammary gland of severe combined immunodeficiency (SCID) beige or nude mice respectively, blocking the CCL2-CCR2 axis with anti-CCL2 antibody can effectively inhibit macrophage recruitment and thus inhibit breast tumor metastasis (107). Leuschner et al. (108) developed liposome NPs loaded with CCR2 siRNA to mediate CCR2 gene silencing in Ly-6C high monocytes. The Liposome NPs improved drug concentration in the tumor and regulated the function of innate immune cell subtypes. TAMs reduced by 54% in the EL4 transplanted tumor model and by 75% in the CT26 transplanted tumor model, respectively. Conde et al. (109) designed a gold nanoparticle (AuNP) based on M2 polypeptide for VEGF siRNA delivery. The AuNPs actively targeted TAMs *in vivo* and passively targeted lung cancer cells. Therefore, AuNPs simultaneously inhibited the VEGF pathway in both cells, causing the reduction of TAM and inhibition of tumor cell proliferation. In mice experiments, the size of lung cancer was reduced by 95% and the survival rate increased by 75%.

### 4.3 Repolarization of M2 macrophages

Although TAMs show mainly the tumor-promoting phenotype M2, TAMs may convert from tumor-promoting M2 to tumor-killing M1, which is called repolarization, at different stages of tumor development and treatment. As an important phagocyte, M1 macrophages can present antigen and inhibit tumor growth by activating the immune response (110). At present, comprehensive studies on macrophage polarization regulation strategies focus on primarily on the regulation of macrophage-specific antibodies or signaling pathways, genetic/epigenetic level regulation of macrophages, and the TME.

#### 4.3.1 Regulation of targeted macrophage-associated receptors

CD40 is a member of the TNF receptor superfamilies expressed on the surface of macrophages. The interaction between CD40 and CD40L can induce macrophages to produce pro-inflammatory cytokines and overexpress MHC molecules. Therefore, treatment with the CD40 agonist can easily activate macrophages to perform tumor killing functions and restore immune surveillance of tumors. Such CD40-activating antibodies CP-870893 and RO7009789 are being evaluated in clinical trials (111, 112).

The collagen-structured macrophage receptor (MARCO) is a pattern recognition receptor belonging to the scavenger receptor family. MARCO is mainly expressed by macrophages. Its high expression is associated with the poor prognosis of many tumors. It is reported that when combined with CTLA-4 checkpoint inhibitors, antibodies against the MARCO receptor can repolarize TAMs to a pro-inflammatory phenotype and improve the antitumor immune response (113).

The Toll-like receptor (TLR) is another PRR which plays an important role in the activation of the innate immune response. The activation of TLR by bacterial particles, such as LPS, or viral nucleic acids, such as RNA or DNA, could initiate an innate immune response and allow macrophages to switch the pro-inflammatory M1 phenotype. Various TLR agonists can mimic microbial signals and convert TAMs into tumor killing phenotypes (114). Toll-like receptor 2 (TLR2) agonists have been reported to specifically stimulate macrophage antitumor potential after intratumoral injection in melanoma models (115). As an agonist of TLR7 and TLR8, requimod (R848) can also induce macrophages to the M1 phenotype. To obtain more accumulation of agonists in the TME after intravenous injection, Weissleder et al. (116) designed R848-loaded β-cyclodextrin NPs (CDNP-R848) that achieved efficient drug delivery to tumor-associated macrophages *in vivo*. R848, an agonist of the toll-like receptors TLR7 and TLR8 identified in a morphometric-based screen, is a potent driver of the M1 phenotype *in vitro*. As monotherapy, the administration of CDNP-R848 in multiple tumor models in mice altered the functional orientation of the tumor immune microenvironment towards an M1 phenotype, leading to controlled tumor growth and protected the animals against tumor rechallenge.

#### 4.3.2 Regulation of macrophage-related signaling pathways

The PTEN/PI3Kγ/mTOR signaling pathway has been reported to regulate the immune microenvironment of tumors through macrophage repolarization during cancer development (117). Li et al. (118) synthesized porous iron oxide NPs (PHNPs) to load a PI3Kγ small molecule inhibitor, 3-methyladenine (3-MA), which is further modified by mannose to target TAM. This delivery system showed good targeting efficiency. The macrophage inflammatory factor NF-κB p65 is activated by the combination of PHNP and 3-MA, which helps to convert TAMs to pro-inflammatory M1 macrophages. The delivery system activated the immune response *in vivo* and inhibited tumor growth. The tumor volume decreased about 64%.

Activation of the interferon gene cyclic GMP-AMP synthase-stimulator of interferon genes (cGAS-STING) pathway produces a variety of pro-inflammatory chemokines and cytokines, such as IFN, to trigger the innate immune response of the body against tumor (119). IFN-I promotes the Th1-mediated immune response, so it can polarize TAMs into the M1 subtype. Sting agonists include cyclic dimeric adenosine monophosphate (c-di-AMP), cyclic dimeric guanosine monophosphate (c-di-GMP), and cyclic GMP AMP (cGAMP) (120). Shae et al. (121) designed STING activating NPs (STING-NP), rationally designed polymersomes to achieve enhanced cytosolic delivery of the endogenous CDN ligand for STING, 2’3’ cyclic guanosine monophosphate-adenosine monophosphate (cGAMP), which is the endogenous and high affinity ligand for STING. Using the B16F10 melanoma transplantation model in immunocompetent mice, a single intratumoral treatment with STING-NPs, NPs decreased expression of CD206, a typical marker of M2 macrophages, expressed on the surface of macrophages, which suggested repolarization or recruitment of macrophages with reduced immunosuppressive capacity. Compared to free cGAMP, STING-NPs increased the expression of interferon-β1 by 6.3 times, increase Cxcl9 by 6.6-fold and Cxcl10 by 4.9-fold, indicating that STING-NPs increased the immunostimulatory potency of 2’3’-cGAMP. STING-NPs also significantly increased the number of infiltrating CD8 and CD4 T cells in the rat model. The nano-assemblies using Mn^2+^ have also been proven to act as a STING agonist to improve cancer therapy, and activates the immune system. Mn^2+^ coordinated with CDN STING agonists self-assemble into a nanoparticle (CDN^-^Mn^2+^ particle, CMP) that can effectively deliver STING agonists to immune cells. CT26 tumor-bearing BABL/c mice were treated intravenously on days 9, 12, and 15 with 20 μg CDA and 10 μg Mn2+ either in CMPCDA or soluble form. CMP_CDA_ administered intravenously significantly decreased CT26 tumor growth and eliminated established tumors in 50% of mice (P< 0.0001), whereas treatment with soluble CDA^ +^ Mn^2+^ had a 0% response rate (122). Song et al. (123) developed mannan conjugated MnO_2_ particles modified with hyaluronic acid (HA) (Man-HA-MnO_2_), reprogrammed anti-inflammatory and tumor promoting M2 TAM into pro-inflammatory and anti-tumor M1 tumor macrophages. The 4T1 murine breast cancer model was established by subcutaneous injection of 106 cells/mL of 4T1 cells into the flank of BALB/C female mice. The tumor showed 50.3% less tissue hypoxia, 49.3% decrease in expression of HIF-1α, and 31.8% decrease in VEGF expression 4 days after intravenous treatment with 13.2 mg/kg Man-HA-MnO2 NPs compared to those in the saline treatment group, indicating the ability of Man-HA-MnO2 NP to reduce tumor hypoxia. Tumors were collected and subjected to immunostaining with CD206 and IL-10 M2 macrophage markers and iNOS and IL-12 M1 macrophage markers at 48 h after injection. Similar to the *in vitro* results, the percentage of M2 macrophages in tumors decreased, while that of M1 macrophages increased.

We have discussed protein or molecular changes thus far. However, macrophage function is regulated through the surface receptor or via the intracellular signaling pathway of the macrophage by antibodies or small molecule drugs. It is also possible to directly regulate the transcriptional expression of cells and adjust cell function by delivering nucleotides into cells at the nucleic acid level. In addition, TME regulation can also alter macrophage function.

#### 4.3.3 Genetic or epigenetic regulation

Repolarization of TAMs could be induced at the genetic and epigenetic levels. As an important regulator of transcription and translation, active miRNA has a significant effect on TAM polarization. Some miRNAs have been reported to be rapidly upregulated by TLR ligands in macrophages, which can regulate macrophage activation and function in tissues, including miR-155, mir-125a/b, and miR-146a (124). Liu et al. (125) designed redox/pH dual-responsive redox/pH hybrid polypeptide nanovector consisting of self-crosslinked redox-responsive NPs based on galactose-functionalized n-butylamine-poly(l-lysine)-b-poly(l-cysteine) polypeptides (GLC) coated with DCA-grafted sheddable PEG-PLL (sPEG) copolymers. The *ex vivo* study revealed that the sPEG shielded cationic GLC core at physiological pH but was quickly shed to re-expose the GLC because of its charge reversible property. PEG/GLC nanovectors effectively facilitate macrophage-targeted miR delivery under acidic conditions, but decreased miR uptake at a neutral pH. Administration of miR155-loaded sPEG/GLC nanocomplexes (sPEG/GLC/155) increased miR155 expression in TAM 100–400 times both *in vitro* and *in vivo*. sPEG/GLC/155 also effectively repolarized immunosuppressive TAMs to antitumor M1 macrophages by increasing M1 macrophage markers (IL-12, iNOS, MHC II) and suppressing M2 macrophage markers (Msr2 and Arg1) in TAM. Furthermore, the treatment of sPEG/GLC/155 significantly increased activated T lymphocytes and NK cells in tumors, which consequently led to robust tumor regression

#### 4.3.4 Regulation of the tumor microenvironment

Some inorganic NPs, such as iron oxide NPs and calcium carbonate NPs, can mediate the repolarization of TAM by altering reactive oxygen species and the acidity of TME (126). Zanganeh et al. (127) reported that iron oxide NPs inhibited tumor growth by inducing the polarization of pro-inflammatory macrophages in tumor tissue. Adenocarcinoma cells co-incubated with ferumoxytol and macrophages *in vitro* resulted in an increase in caspase-3 activity, while macrophages exposed to ferumoxytol showed an increase in mRNA associated with the pro-inflammatory Th1 response. Ferumoxytol significantly inhibited the growth of subcutaneous adenocarcinoma in mice with an inhibition rate of 57%. Chen et al. (126) developed an *in situ* immunotherapeutic bioresponsive gel that contained CaCO3 NPs loaded with anti-CD47 antibody and (aCD47@CaCO_3_). The bioresponsive gel liminated H^+^ in the tumor, allowing polarization of TAMs to the M1 phenotype. Likewise, the released anti-CD47 antibody blocked the “Do not eat me” signal in cancer cells, thus increasing macrophage phagocytosis of cancer cells. Tumor volume decreased by 95% and the animal survival increased by 50%.

### 4.4 Enhancement of phagocytosis

Enhanced M1 phagocytosis can be achieved by stimulating calreticulin or phosphatidylserine in tumor cells, or by blocking the CD47/SIRPα (“Do not eat me”) signaling axis (128). For example, pharmacological inhibition of this signaling pathway by anti-CD47 monoclonal antibody or by the soluble recombinant fusion protein SIRP α-FC can effectively increase TAM phagocytosis in tumor cells (129). Recently, Nie et al. (130) synthesized pH-responsive M1 exosome nanobioconjugates for cancer treatment, in which azide-modified M1 exosomes conjugated with dibenzo cyclooctyne-modified anti-CD47 and anti-SIRPα antibody (aCD47 and aSIRPα). After intravenous administration, nanobiological conjugates were actively concentrated in the tumor through the specific recognition of aCD47 and CD47 on the surface of tumor cells. The acidic environment of the tumor facilitated cleavage of the benimide bond and released aSIRPα and aCD47 in macrophages. The “Do not eat me” signal decreased and macrophage phagocytosis was restored. Meanwhile, M1-derived exosomes effectively repolarized macrophages from M2 to M1. Tumor volume decreased by more than 70%.

An increasing number of studies have shown that macrophages exert an important effect on tumor development, metastasis, immune regulation, tumor angiogenesis, TME remodeling, and cancer treatment. Macrophage targeted therapy is promising and will be the next frontier in tumor immunotherapy.

## 5 The state of the art of DDSs

Nanometer DDSs targeting T cells, NK cells, and TAMs have achieved inspiring results and have become an important strategy to improve the efficacy of immunotherapy. Nanometer drug carriers have unique advantages. Firstly, the small size of NPs will increase drug accumulation at the target site with the help of an enhanced permeability and retention (EPR) effect at the tumor site. Second, the structure of NPs can improve the pharmacological properties of therapeutic drugs, by reducing the toxicity of the encapsulated drugs, prolonging the period of drug circulation, and improving accumulation at the target site. Third, the surface of NPs can be easily modified to achieve targeted drug delivery by cross-linking active targeting ligands. Fourth, NPs with a large specific surface area and high drug loading capacity can achieve multi-drug delivery, improve therapeutic efficacy, and favor complementary roles of different drugs. Fifth, NPs are versatile, allowing different modifications, and thereby can respond to a variety of internal stimuli, such as pH, temperature, enzymes, and redox potential, and external stimuli, such as magnetism, light, and ultrasound, to trigger drug release.

NPs can be divided into organic and inorganic NPs according to their composition and properties. Organic NPs include liposomes, lipid NPs, micelles, and dendrimers. Lipid NPs (LNPs) are particles with a homogeneous lipid core that are widely used for targeted delivery. LNPs have recently gained great attention for their success as a delivery platform for COVID-19 mRNA vaccines (131). Inorganic NPs mainly include metal NPs, mesoporous silica NPs, and quantum dots. Inorganic NPs are usually smaller in size and narrower in distribution than liposomal nanoparticles, with surfaces suitable for ligand coupling (132). AuNPs are stable and have been widely used for research and development due to their inherent optical properties and ease of chemical modification on the surface with multiple types of ligands. AuNPs have attracted great attention in nucleic acid delivery (133, 134). Mesoporous silica NPs (MSN) have many properties, such as being uniform in size, porous, dispersible, biocompatible, and have low toxicity. Its huge surface area makes it a potential excellent nano-carrier (135). The size, shape, and surface of NPs affect their immunomodulatory effects. Foged et al. (136) reported that the optimal particle size for human DC cell uptake is below 500 nm. Smaller NPs (20-200 nm) could be internalized into DCs and macrophages located in lymph nodes. They could diffuse and spread independently in peripheral lymph nodes, whereas larger NPs (500–2000 nm) were taken up by peripheral APCs at the injection site, requiring diffusion-based transport. The shape of NPs also affected their immunogenic effects. In comparison to the immunogenicity of spherical, cubic, and rod-shaped gold NPs, the most efficient APC uptake was found in rod-shaped NPs. Instead, 40 nm spherical NPs were able to increase pro-inflammatory cytokine secretion as superior delivery platforms (137). The surface charge of the NPs had a significant impact on their internalization. Negatively charged cell membranes increased the affinity for positively charged NPs. Compared with negatively charged NPs, positively charged NPs enhanced the absorption of DCs through electron binding (138). In addition, by crosslinking receptor-targeting molecules to particles, surface-functionalized particle carriers were fabricated to improve antigen presenting, immune activation or cancer cell killing. Monoclonal antibody drugs, including ICIs, have made great success in cancer immunotherapies. Whereas, they are also brilliant targeting molecules in the development of nanoparticle-based DDSs. The current hot targets on T cells, NK cells and TAMs and corresponding representative antibody drugs that have been approved to market or in clinical studies were summarized in **Table 1**.

**Table 1:** Hot targets and representative antibodies.

Hot targets and representative antibodies			
Target		Antibody therapeutics products	Antibodies in late-stage clinical studies
T cell	CTLA-4	Tremelimumab, Ipilimumab,	Zalifrelimab, Quavonlimab, Ipilimumab biosimilar, Tuvonralimab, Erfonrilimab
	PD-1	Pembrolizumab, Nivolumab, Toripalimab, Tislelizumab, Sintilimab, Serplulimab, Retifanlimab, Pucotenlimab, Prolgolimab, Penpulimab, Geptanolimab (Genolimzumab), Dostarlimab, Cemiplimab (cemiplimab-rwlc), Camrelizumab, Enlonstobart, Cadonilimab,	Sasanlimab, Rulonilimab, Nofazinlimab, Finotonlimab, Cetrelimab, QL1604, zimberelimab, Tebotelimab, Iparomlimab, Ivonescimab
	PD-L1	Tagitanlimab, Sugemalimab, Socazolimab, Envafolimab, Durvalumab, Cosibelimab, Avelumab, Atezolizumab, Adebrelimab	APL-502, Erfonrilimab, Bintrafusp alfa, Retlirafusp alfa
	CD40L	\	Dapirolizumab pegol,dazodalibep,Letolizumab (BMS-986004),VIB4920
	LAG-3	relatlimab	Fianlimab, Favezelimab, Tebotelimab, TSR-033, BI-754111, LAG525, GSK2831781, INCAGN2385-101, DNV3, MK-4280
	OX40	\	Rocatinlimab, Ivuxolimab, PF-04518600, IBI101, BMS-986178, MEDI-6383, ABBV-368
	TIGIT	\	Vibostolimab, Tiragolumab, Ociperlimab, Domvanalimab, etigilimab
	TIM-3	\	Sabatolimab, Cobolimab, BMS-986258, INCAGN-02390
	CD27	\	Varlilumab, MK-5890
	CD70	\	ARGX-110, MDX1411, SEA-CD70
	BTLA	\	icatolimab, HFB200603
	GITR	\	BMS-986156, ASP1951(PTZ-522), MEDI1873
	4-1BB	\	ADG106, Urelumab,Utomilumab
	CD40	\	YH-003, BSI-038, CDX-1140, SEA-CD40, ADC-1013, APX005M
	ICOS	\	feladilimab, MEDI-570, Vopratelimab
	Multispecific antibody	Cadonilimab	CDX-527,TJ-L14B/ABL503,GNC-038,ZGGS15,MGD-013,FS118,XmAb22841,RO 7121661 (RG 7769),AZD-7789,XmAb23104,GEN-1046,ND-021,GNC-035, KN046
NK cell	CD16A	\	AFM24, AFM12, AFM13
	CD16a-IL-15/CD33	\	GTB-3550
	NKp30	\	CTX-8573
	5T4/CD16/NKG2D	\	DF7001
	KIR3DL-2	\	Lacutamab (IPH4102)
	NKG2A	\	monalizumab, BMS-986315
	NKp46	\	Cytovia,INNATE PHARMA
	NKp46/CD16	\	SAR443579, IPH62
	NKp46/CD38	\	CYT-338
	CD19/CD3	blinatumomab	\
	HER2/CD3/CD28	\	SAR443216
Macrophage	CD47	\	Letaplimab, IBI-322, magrolimab, SRF231, ALX148, AO-176
	SIRPα	\	OSE-172, TTI-622, TTI-621, CC95251
	CSF-1R	\	emactuzumab, PLX-3397, PLX-7486, ARRY-382, AMG820, FPA008
	TREM1	\	PY-159
	TREM2	\	AL-002, PY-314
	PSGL-1	\	SSGJ-617

\, None.

Based on the rapid development of NP engineering, bionic NPs further broaden the applications of nanocarriers-mediated targeting immunotherapy. Bionic NPs can mimic the structure, function, and biosynthetic pathways of the biological system, exhibiting high biocompatibility, low immunogenicity, long systemic circulation, and focal targeting (139–141). Bionic NPs have stronger targeting and immune-activating functions. As drug delivery carriers, different bionic NP formulations have improved therapeutic efficacy by selectively delivering drugs to targeted cells or to the TME. Leukocytes are important immune cells in the body that recognize inflammation and selectively accumulate toward inflamed tissues. Since chronic inflammation is one of the main clinical features of cancer (142), inflammatory tropism can be utilized by preparing nanocarriers wrapped around leukocyte membranes, and targeted tumor delivery can be achieved. Platelets can be used in the targeted treatment of vascular damage caused by a variety of factors. Vascular leakage or inflammatory responses during cancerous processes stimulates platelet adhesion to form tumor thrombi, which in turn helps cancerous cells evade recognition by the immune system and attack by NK cells. Taking advantage of the high colocalization of tumor cells and platelets, platelet-loading NPs can be used for tumor-targeted delivery as drug carriers.

Various autologous cells are also natural and excellent platforms for circulating delivery (78, 143–145). Circulating cells, such as erythrocytes, which are highly mobile, flexible, loaded with high drug-loading, and stealthy, can evade from a recognition by the autoimmune system. Immune cells, such as T cells, NK cells, and macrophages, possess both characteristics of circulating cells and immunological characteristics, such as tumor infiltration and tumor tropism. As drug delivery carriers, they perform both targeting delivery and immune effects. In addition to pathogenicity, tumor cells are also the most ideal drug carriers to penetrate the tumor. Modified tumor cells do not have pathogenicity, but retain stealth, which allows cells to avoid recognition of the autoimmune system and the defense mechanism of tumor cells (146). Taking advantage of ‘homing properties’ and autoimmune agent specificity, they can achieve the targeted delivery of anti-tumor drugs or use apoptotic receptors to induce tumor cell death and achieve the desired therapeutic effect.

In summary, theoretical and technological advances in various disciplines have facilitated the development of novel DDSs. The intense research on the immune system has led to the discovery of an increasing number of promising new targets and new signaling pathways for cancer immunotherapy. Advances in biomaterials have driven the development of DDSs, and bionic nanomaterials have led to advances in artificial biomimetic NPs, synthetic cell delivery platforms, and cellular carriers. Bioengineering has made it possible to customize specific monoclonal and polyclonal antibodies and has also promoted the development of cellular and viral vectors. A new chapter in tumor immunotherapy has been opened by these technological advances. However, because the complexities of the cellular context, such as the complex dynamic network of receptor ligands, the crosstalk between different molecular pairs, genetic diversity, the wide range of receptor expression, low drugloading or drug release before reaching the target site, nonspecific cytotoxicity, and cell mapping, cancer immunotherapy is complex and requires multiple combination strategies. Combining therapeutic strategies puts greater demands on DDSs, which prompts us to integrate multidisciplinary technologies to design safer, more efficient, more accurate, and more intelligent multifunctional DDSs to continuously improve cancer immunotherapy.

## Author contributions

Conceptualization, F-hX; investigation, Y-lY, FY, Z-qH, QS, and SY; resources, YL and H-yS; writing-original draft preparation, Y-lY, FY, Z-qH, QS, and YM; writing-review and editing, Y-lY and F-hX; visualization, Y-lY; proof-reading, YW, YZ, and G-rZ; supervision, F-hX and G-rZ; project administration, F-hX. All authors contributed to the article and approved the submitted version.

## Glossary

**Table:**

(NK)	natural killer
(TAMs)	tumor associated macrophages
(DDS)	drug delivery system
(CTLs)	cytotoxic T lymphocytes
(DAMPs)	damage associated molecular patterns
(PRRs)	pattern recognition receptors
(APCs)	antigen-presenting cells
(DCs)	dendritic cells
(TNF)	tumor necrosis factor
(CAR)-engineered T cells	chimeric antigen receptor
(CAR)-engineered T cells (CAR-T)	chimeric antigen receptor
(TCRs)	T cell receptors
(MHC)	major histocompatibility complex
(PD-1)	programmed cell death protein 1
(PD-L1)	programmed cell death ligand 1
(FasL)	Fas ligand
(CD95L)	FasL
(CD95)	Fas
(IFN)	interferon
(CTLA-4)	cytotoxic T-lymphocyte antigen 4
(aAPC)	artificial APCs
(pMHC-I)	peptide-loaded major histocompatibility complex Class I
(αCD28)	anti-CD28
(naAPCs)	nanodimensional artificial antigen presenting cells
(CHA)	chlorogenic acid
(SMEDDS)	self-microemulsifying drug delivery systems
(STING)	stimulator of interferon genes
(MUSIC)	microbubble-assisted ultrasound-guided immunotherapy of cancer
(cGAMP)	cyclic guanosine monophosphate-adenosine monophosphate
(TNFRSF5)	tumor necrosis factor receptor superfamily member 5
(TNFRSF9)	tumor necrosis factor receptor superfamily member 9
(TRAF)	TNF receptor-associated factor
(MAPK)	mitogen-activated protein kinase
(IL-2)	interleukin 2
(Gal-1)	galectin-1
(VISTA)	V-domain Ig suppressor of T cell activation
(TIGIT)	T cell immunoreceptor with immunoglobulin and ITIM domains
(GITR)	glucocorticoid-induced TNFR-related protein
(TNFRSF4)	tumor necrosis factor receptor superfamily member 4
(PD-L2)	programmed cell death ligand 2
(CpG-ODN)	cytosine-phosphate-guanosine oligodeoxynucleotides
(ssDNA)	single-stranded DNA
	nanoparticles (NPs)
(TGMS) nanoparticles	triglycerol monostearate
	(TGMS NPs)
(MMPs)	matrix metalloproteinases
(TME)	tumor microenvironment
(GBM)	glioblastoma multiforme
(TNFRSF18, CD357)	tumor necrosis factor receptor superfamily 18
(ESCC)	esophageal squamous cell carcinoma
(PLGA)	Poly (lactic-co-glycolic acid)
(ITAM)	immunoreceptor tyrosine-based activation motif
(LFA-1)	lymphocyte function-associated antigen-1
(Mac-1)	macrophage-1 antigen
(VLA-4; CD49d/CD29)	integrin very late antigen-4
(TNF)-related apoptosis-inducing ligand	tumor necrosis factor
	(TRAIL)
(GSDMB)	gasdermin B
(NKSF)	Natural killer cell stimulatory factor
(TGF-β)	transforming growth factor-β
(PG)	prostaglandins
(KIRs)	killer immunoglobulin-like receptors
(ICI)	immune checkpoint inhibitors
(TIM-3)	T cell immunoglobulin and mucin-containing molecule 3
(LAG-3)	lymphocyte activation gene-3
(EGFR)	epidermal growth factor receptor
(AML)	acute myeloid leukemia
(nano-TriNKE)	nanoparticle-based trispecific NK cell engager
(PEG-PLGA)	Poly (ethylene glycol)–block-poly(lactide-co-glycolide)
(NKCE)	NK cell engager
(HR-MDS)	high risk-myelodysplastic syndrome
(oNK)	oNK cell line
(BiKE)	NK cell-targeted bispecific antibodies
(MRD)	minimal residual disease
(NSCLC)	non-small-cell lung cancer
(CSF-1)	Colony stimulating factor 1
(LPS)	Lipopolysaccharide
(GM-CSF)	granulocyte-macrophage colony-stimulating factor
(ROS)	reactive oxygen species
(NO)	nitric oxide
(Th1)	type 1 helper T
(HIF)	hypoxia inducible factor
(VEGF)	vascular endothelial growth factor
(FRβ)	Folate receptors β
(AuNPs)	gold nanoparticle
(MARCO)	macrophage receptor with collagen structure
(TLR2)	toll-like receptor 2
(cGAS-STING)	the cyclic GMP-AMP synthase-stimulator of interferon genes
(cGAMP)	2’3’ cyclic guanosine monophosphate-adenosine monophosphate
(EPR)	enhanced permeability and retention
